# Optimized Construction of Highly Efficient P-Bi_2_MoO_6_/g-C_3_N_4_ Photocatalytic Bactericide: Based on Source Material and Synthesis Process

**DOI:** 10.3390/nano15110834

**Published:** 2025-05-30

**Authors:** Leilei Xue, Jie Zhang, Mengmeng Sun, Hui Zhang, Ke Wang, Debao Wang, Ruiyong Zhang

**Affiliations:** 1 College of Chemistry and Molecular Engineering, Qingdao University of Science and Technology, Qingdao 266042, China; xueleilei0731@163.com (L.X.); dbwang@qust.edu.cn (D.W.); 2 State Key Laboratory of Advanced Marine Materials, Key Laboratory of Marine Environmental Corrosion and Bio-Fouling, Institute of Oceanology, Chinese Academy of Sciences, Qingdao 266071, China; 3 University of Chinese Academy of Sciences, 19 (Jia) Yuquan Road, Beijing 100049, China

**Keywords:** Bi_2_MoO_6_, g-C_3_N_4_, photocatalytic sterilization, doping

## Abstract

In this study, Bi_2_MoO_6_ nanoflowers with different molybdenum sources were in situ grown on the surface of g-C_3_N_4_ nanosheets (OCN) by a simple one-step solvothermal method. The effects of doping and different molybdenum sources on the photocatalytic degradation and bactericidal activity of Bi_2_MoO_6_/OCN were discussed. Among them, the solvothermal preparation of P-Bi_2_MoO_6_/OCN using phosphomolybdic acid as molybdenum source can make up for the shortcomings caused by the destruction of OCN structure by generating more lattice defects to promote charge separation and constructing Lewis acid/base sites to effectively improve the photocatalytic performance. In addition, by adding phosphoric acid to increase the P-doped content, more exposed alkaline active sites are induced on the surface of P-Bi_2_MoO_6_/OCN, as well as larger specific surface area and charge transfer efficiency, which further improve the photocatalytic performance. Finally, the optimized 16P-Bi_2_MoO_6_/OCN showed a degradation rate of 99.7% for 20 mg/L rhodamine B (RhB) within 80 min under visible light, and the antibacterial rates against *E. coli*, *S. aureus* and *P. aeruginosa* within 300 min were 99.58%, 98.20% and 97.48%, respectively. This study provides a reference for optimizing the synthesis of environmentally friendly, solar-responsive, photocatalytic sterilization materials from the perspective of preparation, raw materials and structure.

## 1. Introduction

The threat of microbial contamination to human health and the economy has attracted more and more attention in many fields. In addition to daily life and medical disinfection, the key role of bacteria in the biological pollution of water environments such as oceans has also prompted the development of new antibacterial and antifouling methods. Although traditional antibacterial materials have effective bactericidal effects, the production cost of many materials is high. The heavy metal ions and toxic substances exuded during the action process can easily cause secondary pollution to the environment and are also prone to biological resistance, laying hidden dangers for long-term prevention and control. Looking for green, efficient and low-cost antibacterial and anti-fouling methods has become an important issue that is closely related to social development and people’s life safety and cannot be ignored.

In recent years, photocatalytic technology has attracted more and more attention in the fields of green energy, environmental management, pollution control and medical and health care [1,2,3]. Its unique photo-induced redox reaction mechanism has wide applicability, and the controllability of photocatalytic materials also provides great potential for research and development. At present, the most studied photocatalytic materials are TiO_2_ [4], graphitic carbon nitride (g-C_3_N_4_) [5], metal-organic frameworks (MOFs) [6], MXene [7], etc., which are widely used in the photocatalytic decomposition of water to produce hydrogen, degradation of dyes or antibiotics, photocatalytic nitrogen fixation and CO_2_ conversion. With the development of the marine economy, the application potential of photocatalytic technology in the field of prevention and control of biological fouling has also begun to receive attention. However, the development of photocatalytic materials is still limited by the shortcomings of low separation efficiency and easy recombination of photogenerated carriers [8,9,10]. It is still a huge challenge to develop efficient and stable new photocatalytic materials suitable for various fields.

g-C_3_N_4_ is a non-metallic, two-dimensional semiconductor material with excellent stability, environmental protection performance and low preparation cost. As a photocatalytic material, it also has an appropriate band gap and a wide light response range. The modification of pure g-C_3_N_4_ is often carried out by adjusting the morphology. For instance, g-C_3_N_4_ with special morphologies, such as porous, single-layer nanosheets, hollow spheres and nanotubes, is designed and synthesized [11,12,13]. Ultimately, g-C_3_N_4_ with larger specific surface area, smaller pore size and higher crystallinity is prepared. In this regard, more exposed active sites are the key to improving photocatalytic performance. However, subject to the disadvantages of slow charge separation and transport and easy recombination of free carriers, g-C_3_N_4_ is more suitable for constructing heterojunctions with other materials to obtain more efficient photocatalytic performance.

In addition to typical catalytic materials such as TiO_2_, MOFs and ZnO_2_, non-toxic bismuth-based photocatalysts such as Bi_2_WO_6_ [14], BiVO_4_ [15,16] and Bi_2_MoO_6_ [17,18] are also regarded as environmentally friendly, non-toxic photocatalysts and have received extensive attention. Bi_2_MoO_6_ is an Aurivillius-type oxide formed by alternating layers of [MoO_4_]^2−^ and [Bi_2_O_2_]^2+^, which are connected by oxygen atoms [19]. Similarly to g-C_3_N_4_, its non-toxicity, cost-effectiveness and broad-spectrum light response make it suitable as an environmentally friendly photocatalytic antifouling material. However, similarly, the photocatalytic performance of Bi_2_MoO_6_ monomer is also not good, due to its low quantum yield and rapid recombination of photogenerated carriers [20,21]. The modification of Bi_2_MoO_6_ monomer is mainly to improve the photocatalytic efficiency by doping and inducing lattice defects. The different doping elements may affect the band structure, charge transfer and surface adsorption of the catalyst. Chang et al., by doping In^3+^, directly replaced Bi^3+^ ions in the [Bi_2_O_2_]^2+^ layer, resulting in surface trapping traps to reduce the recombination rate of photogenerated carriers, causing the lattice compressive strain to induce the formation of a local built-in electric field to improve the charge transfer efficiency, so that the optimized Bi_2_MoO_6_ exhibits enhanced photocatalytic degradation of antibiotics [22]. By doping Fe^3+^ to partially replace Bi^3+^, Li et al. enhanced the asymmetry inside the Bi_2_MoO_6_ lattice, improved the surface separation efficiency of photogenerated electron–hole pairs and significantly improved the photocatalytic dye degradation and water splitting oxygen production capacity [23]. However, in previous studies on the doping modification of Bi_2_MoO_6_, although sodium molybdate dihydrate or ammonium molybdate tetrahydrate was used as the molybdenum source to prepare Bi_2_MoO_6_ photocatalyst, the effect of introducing Na and N doping from the reaction source on the photocatalytic performance of the system was rarely discussed. In order to design and construct a better Bi_2_MoO_6_-based photocatalytic material, it is obviously necessary to compare and analyze the effects of doping atoms introduced by different molybdenum sources in detail. In this paper, phosphomolybdic acid (H_3_PO_4_·12MoO_3_) is selected as the optimized molybdenum source. This is because P doping often associates to produce acidic and alkaline active centers, which can interact with g-C_3_N_4_ with strong Lewis bases to form sterically hindered Lewis bases and Lewis acids, which greatly enhances the adsorption and activation of reactant molecules [24]. At the same time, previous studies on Bi_2_MoO_6_ and g-C_3_N_4_ composites have also shown that they have a suitable band gap structure and can form type I [25], type II [26], type Z [27] and type S [28,29] heterojunctions to construct new carrier transfer channels to promote separation.

It cannot be ignored that for the composite materials of the doped system, due to the dual effects of doping modification and composite modification, the influencing factors are more complex, and it is difficult to obtain the target material with improved performance. In this paper, the Bi_2_MoO_6_/g-C_3_N_4_ system is taken as an example to analyze and explore this complex process in detail. Firstly, Bi_2_MoO_6_ nanoflowers with different molybdenum sources were in situ grown on the surface of g-C_3_N_4_ nanosheets (OCN) by a simple one-step solvothermal method under the same conditions. P-Bi_2_MoO_6_/OCN (phosphomolybdate as molybdenum source), Na-Bi_2_MoO_6_/OCN (sodium molybdate as molybdenum source) and N-Bi_2_MoO_6_/OCN (ammonium molybdate as molybdenum source) heterojunction composites were obtained. The effects of different molybdenum source doping on the structure and photocatalytic performance of Bi_2_MoO_6_/g-C_3_N_4_ heterojunction were studied, and the potential reaction mechanism was analyzed. After solvothermal treatment, large-area OCN nanosheets shrank into small-area interlaced g-C_3_N_4_ nanosheets, and the specific surface area and active sites were reduced. After comparing the photocatalytic degradation performance of dyes under visible light, it was found that although the heterojunction of Bi_2_MoO_6_ doped with Na, N and contracted OCN could effectively inhibit the recombination of photogenerated carriers to a certain extent, the photocatalytic performance was still lower than that of pure OCN due to the inability to strongly compensate for the reduction of contracted OCN active sites. The difference was that the P-doped Bi_2_MoO_6_/g-C_3_N_4_ system prepared with phosphomolybdic acid as the molybdenum source effectively compensated for the unfavorable factors caused by the structural damage of OCN by generating more Bi vacancy lattice defects to capture holes and introducing highly active reaction sites, such as Lewis acid/base sites, and significantly improved the photocatalytic activity. The degradation rate of 20 mg/L RhB was 98.8% under visible light for 120 min, and the antibacterial rates against *E. coli*, *S. aureus* and *P. aeruginosa* were 93.09%, 92.81% and 91.13% within 300 min, respectively. For further optimization, an appropriate amount of phosphoric acid was added to increase the content of phosphorus doping, induce more [Bi_2_O_2_]^2+^ layer lattice bismuth to form vacancy defects and cover the surface with oxides such as metal Bi and Bi_2_O_3_ to form impurity levels to promote charge separation, with faster charge-transfer efficiency. At the same time, there were more alkaline active sites on the surface of OCN, and the synergistic effect improved the photocatalytic performance. Finally, the optimized 16P-Bi_2_MoO_6_/OCN showed a degradation rate of 99.7% for 20 mg/L RhB within 80 min under visible light, and the antibacterial rates against *E. coli*, *S. aureus* and *P. aeruginosa* within 300 min were 99.58%, 98.20% and 97.48%, respectively. This study provides a valuable reference for the synthesis of environmentally friendly, solar-responsive, photocatalytic sterilization materials from the perspective of reaction source selection and optimization of the structure of complex systems.

## 2. Experimental Section

### 2.1. Preparation of OCN

A certain amount of urea was put into an alumina crucible, which was wrapped with tin foil, heated from room temperature to 550 °C at 5 °C/min and kept for 2 h in a muffle furnace. After cooling, it was not necessary to take out the crucible. Then, the muffle furnace was heated to 500 °C at the same speed and kept for 2 h. Then, the light-yellow g-C_3_N_4_ was obtained and labeled as OCN.

### 2.2. Preparation of Three Types of Nanostructured Flower-like Bi_2_MoO_6_ Particles

For P-Bi_2_MoO_6_, Na-Bi_2_MoO_6_ and N-Bi_2_MoO_6_, bismuth nitrate pentahydrate (0.485 g) was used as the Bi source, while phosphomolybdate (0.076 g), sodium molybdate dihydrate (0.121 g) and ammonium molybdate tetrahydrate (0.0883 g) were utilized as the Mo sources for the synthesis of Bi_2_MoO_6_ nanoflower particles.

Initially, the aforementioned reactants were dissolved in 5 mL of ethylene glycol and ultrasonically treated for 5 min to ensure thorough dissolution. Subsequently, the Bi source and Mo source were mixed evenly before being slowly added to 20 mL anhydrous ethanol with continuous stirring for 1 h. The resulting mixture was then transferred into a reaction vessel and maintained at 160 °C for a duration of 10 h. Following cooling, it underwent washing with anhydrous ethanol, centrifugation, drying at 60 °C and subsequent grinding into powder. Based on their respective Mo sources representing different doping types, they were denoted as P-Bi_2_MoO_6_, Na-Bi_2_MoO_6_ and N-Bi_2_MoO_6_.

### 2.3. Preparation of Doped-Bi_2_MoO_6_/g-C_3_N_4_ Heterojunction

A total of 0.3 g of OCN was added to 20 mL of anhydrous ethanol, and the g-C_3_N_4_ ethanol dispersion was obtained by ultrasonication for 30 min. The Bi source and Mo source were mixed and slowly added to the g-C_3_N_4_ ethanol dispersion, and the other steps were the same as those used to prepare Bi_2_MoO_6_. According to the mass ratio of g-C_3_N_4_:Bi_2_MoO_6_ = 1:2, OCN composites with three different doped Bi_2_MoO_6_ nanoflower spheres were prepared, labeled as P-Bi_2_MoO_6_/OCN, Na-Bi_2_MoO_6_/OCN and N-Bi_2_MoO_6_/OCN. In addition, in order to optimize P-Bi_2_MoO_6_/OCN heterojunction, a small amount of phosphoric acid was added to the mixture and stirred together for 1 h before the solvothermal reaction. It was named according to the amount of phosphoric acid added. For example, P-Bi_2_MoO_6_/OCN with 16 μL phosphoric acid was named 16P-Bi_2_MoO_6_/OCN.

### 2.4. Characterization of the Materials

Sample characterization methods were as follows: The morphology and structure of the samples were characterized by scanning electron microscopy (SEM, Hitachi Regulus8100, Tokyo, Japan), energy dispersive spectrometer (Thermo Fisher Ultradry Series Noran System7, MA, USA), transmission electron microscopy (TEM, Hitachi HT7700, Tokyo, Japan) and high-resolution transmission electron microscopy (HRTEM, JEM-2100F, NEC Co., Tokyo, Japan). The X-ray diffraction (XRD) patterns were recorded on a Riken Smart Lab diffractometer. The X-ray photoelectron spectroscopy (XPS) results were recorded on a Thermo ESCALAB 250 Xi instrument (Thermo, MA, USA). At the liquid nitrogen temperature (77 K), the Brunauer–Emmett–Teller (BET) specific surface area of the samples was determined by a nitrogen adsorption apparatus (Micromeritics ASAP 2460, GA, USA). The pore size distribution was calculated from the desorption branch of the isotherm according to the Barrett–Joyner–Halenda (BJH) model. Photoluminescence (PL) analysis was performed on a F-4700 (Hitachi, Tokyo, Japan) fluorescence spectrophotometer. The Fourier-transform infrared (FT-IR) spectra were collected with a Thermo Fisher IS5 spectrometer (MA, USA), with KBr as the diluent. CO_2_ and NH_3_ temperature-programmed desorption (CO_2_/NH_3_-TPD) were measured by using a Micromeritics Autochem II2920 temperature programming detector, USA. Superoxide radicals (·O_2_^−^) and hydroxyl radicals (·OH) generated in the photocatalytic reaction were detected by DMPO in methanol and DMPO in water, respectively, and the corresponding EPR signals were recorded on a Bruker EMXplus spectrometer (Bruker Co., MA, USA).

### 2.5. Photocatalytic Degradation Performance

The photocatalytic degradation experiment was carried out using an 800 W xenon lamp with a light intensity of 80 mW/cm^2^ and a 420 nm filter. Rhodamine B (RhB) was chosen as the pollutant dye for degradation in the photocatalytic experiment, with 25 mg of photocatalyst added to a 50 mL solution containing RhB at a concentration of 20 mg/L. Prior to illumination, the photocatalyst was stirred in the RhB solution for 30 min to achieve adsorption–desorption equilibrium. Subsequently, samples were collected every 30 min, and after removing the catalyst by centrifugation, the absorbance of the residual solution was measured at 554 nm using an ELISA meter (AKTAavant25, Thermo Fisher). The concentration of RhB at each sampling time point was determined based on the absorbance–concentration standard curve of the RhB solution.

### 2.6. Photocatalytic Antibacterial Performance

In the photocatalytic antibacterial experiment, the light intensity was the same as that of the degradation experiment. *Escherichia coli* (*E. coli*), *Staphylococcus aureus* (*S. aureus*) and *Pseudomonas aeruginosa* (*P. aeruginosa*) were selected as the typical strains of broad-spectrum antibacterial, and the plate colony counting method was used to calculate the number of colonies to evaluate the antibacterial effect of each photocatalyst. The single colony was inoculated into the LB liquid medium, which had been killed by bacteria, and cultured at 37 °C for 24 h to obtain the original bacterial solution. Five mL of the original bacterial solution was put into a 10 mL centrifuge tube, centrifuged to remove the LB liquid medium, washed with 0.01 mol/L PBS (pH = 7.4) buffer solution twice. Five mL of PBS buffer solution was added, and the solution was shaken well. A total of 49.5 mL of PBS buffer solution and 0.5 mL of the washed original bacterial solution were added into a 50 mL quartz tube, along with 50 mg of photocatalyst. The blank experiment without photocatalyst was used as the control experiment. After adding the photocatalyst, it was stirred in the dark for 1 h to achieve the adsorption–desorption balance. After illumination, it was sampled every 100 min. One hundred μL of the reaction solution was taken each time, several gradients were diluted with 0.01 mol/L PBS buffer solution according to the series dilution method. Then, 100 μL was taken from the solution with different dilution times to the prepared sterile LB solid medium, and the bacterial solution was evenly smeared on the medium by rolling small glass beads. Finally, the LB solid medium was put into a constant temperature incubator at 37 °C for 24 h. The bacterial concentration was calculated by counting the number of colonies grown on the culture medium, with 30~300 colonies on the culture medium as the best counting range. The survival rate and sterilization rate of each group of bacteria were calculated. Each group of experiments was measured three times in parallel, and the average value was taken as the final result. The survival rate of bacteria was calculated according to the following formula:

(1) $$\mathrm{survival}\mathrm{rate}\;(\%)=\frac{C_{t}}{C_{0}}\times 100\%$$

*C*_0_ is the initial number of colonies, and *C_t_* is the number of colonies at the time of illumination *t*. The antibacterial rate is

(2) antibacterialrate (%)=100%−survivalrate (%)

### 2.7. Photoelectrochemical (PEC) Performance

The photoelectrochemical (PEC) and electrochemical performance of the prepared samples were measured under a three-electrode system using CHI660E electrochemical workstation (Shanghai Chenhua Instrument Co., Ltd., Shanghai, China). The 5 mg powder sample was mixed with isopropanol and 5% Nafion solution to form a uniform and fine slurry, which was then coated on the conductive side of FTO conductive glass (coated area: 1 × 1 cm^2^) and was dried naturally to obtain the working electrode for testing. The PEC performance test used a 300 W xenon lamp light source, and a 420 nm filter was installed on it to filter ultraviolet light. The test position was fixed, and the light intensity of the illuminated surface was adjusted to 100 mW/cm^2^. The Ag/AgCl (saturated KCl) electrode was used as the reference electrode, the platinum plate as the counter electrode, and the FTO conductive glass coated with the sample as the working electrode. The photoelectric response i-t curve was tested by 50 s-switching on and off light. The electrochemical impedance spectroscopy (EIS) of the sample was tested under the illumination condition at open circuit potential over frequency range of 100 kHz~0.1 Hz and at the amplitude of the AC voltage of 5 mV. The Mott–Schottky plot was measured under the potential range of −1.0~1.0 V and the frequency of 1000 Hz in the dark. The electrolyte used for the above measurements was 0.1 M Na_2_SO_4_ aqueous solution.

## 3. Results and Discussion

### 3.1. Analyses of Structure and Surface States

**Figure 1:**
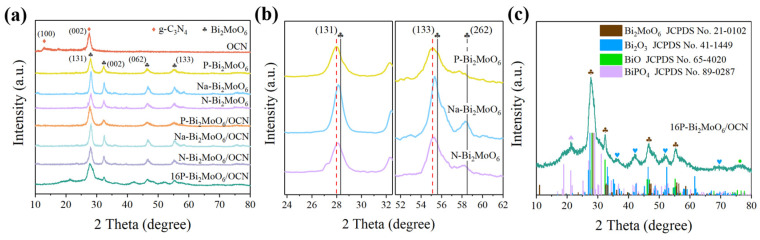
XRD patterns and some details of the as-prepared samples (**a**,**b**); XRD spectrum of 16P-Bi_2_MoO_6_/OCN contains different phases (**c**).

The samples were characterized by XRD to determine the crystal structure and phase details. Figure 1a shows the XRD patterns of three kinds of doped Bi_2_MoO_6_ and their combination with OCN and gives the XRD patterns of pure OCN. OCN exhibits characteristic peaks at about 12.7° and 27.5°, corresponding to the (100) and (002) diffraction planes of graphite-phase carbon nitride, respectively [30]. This is consistent with the crystal plane of the hexagonal g-C_3_N_4_ (JCPDS No. 87-1526). The XRD spectra of pure Bi_2_MoO_6_ prepared by three different molybdenum sources showed diffraction peaks at 27.9°, 32.4°, 46.5° and 55.2°, corresponding to the (131), (002), (062) and (133) crystal planes of orthorhombic bismuth molybdate (JCPDS No.21-0102) [31]. However, compared with the standard Bi_2_MoO_6_ card, the diffraction peaks at (131) and (133) are significantly shifted to a lower angle (Figure 1b), which is due to the increase of the interplanar spacing of Bi_2_MoO_6_ caused by Na, N and P doping. Among them, compared with the XRD spectra of Na-Bi_2_MoO_6_ and N-Bi_2_MoO_6_, P-Bi_2_MoO_6_ has a greater degree of left shift and a greater influence on the interplanar spacing. In addition, compared with the small peak at (262), it is obvious that P doping reduces the crystallinity of Bi_2_MoO_6_ and may introduce more defects. As shown in the Table 1, the calculated cell parameters of several Bi_2_MoO_6_ are not significantly different. Compared to other samples, the unit cell volume of P-Bi_2_MoO_6_ is the largest. The XRD spectra of three kinds of Bi_2_MoO_6_/OCN all show the characteristic peaks of Bi_2_MoO_6_. Due to the overlap of the characteristic peaks of OCN at (002) and Bi_2_MoO_6_ at (131), the decrease of OCN content in the composites, the secondary peak near 12.7° representing the (100) crystal plane of graphite phase carbon nitride becomes less prominent. In contrast, other characterization techniques such as SEM can better show the close bonding between Bi_2_MoO_6_ and OCN. It is worth noting that after adding phosphoric acid to increase the P doping content, taking 16P-Bi_2_MoO_6_/OCN as an example, other characteristic peaks such as Bi_2_O_3_ appeared. As shown in Figure 1c, the 16P-Bi_2_MoO_6_/OCN sample contains four different phases, namely Bi_2_MoO_6_, Bi_2_O_3_, BiO and BiPO_4_, and the main characteristic peaks of each phase are distinguished by color in the figure. The increase of P doping content induces more lattice defects such as Bi vacancies. The free Bi finally tends to combine with oxygen and regenerates other oxides at the layer edge. At the same time, P replaces Mo in the [MoO_4_]^2−^ layer, which is the reason for the formation of BiPO_4_ phase.

**Figure 2:**
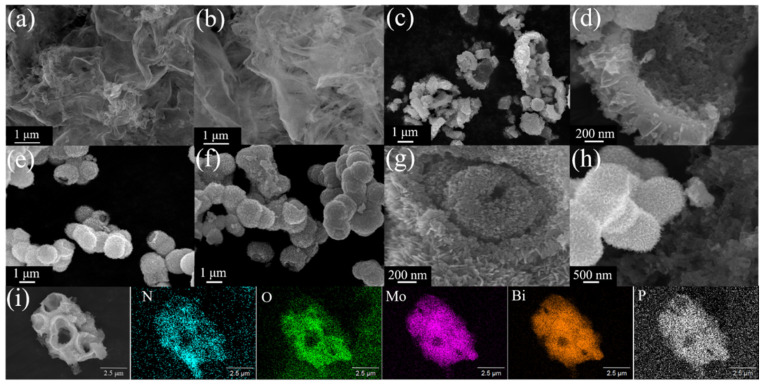
SEM images of OCN (**a**,**b**), P-Bi_2_MoO_6_ (**c**,**d**), Na-Bi_2_MoO_6_ (**e**), N-Bi_2_MoO_6_ (**f**,**g**), P-Bi_2_MoO_6_/OCN (**h**); EDS elemental mapping images of P-Bi_2_MoO_6_/OCN (**i**).

**Figure 3:**
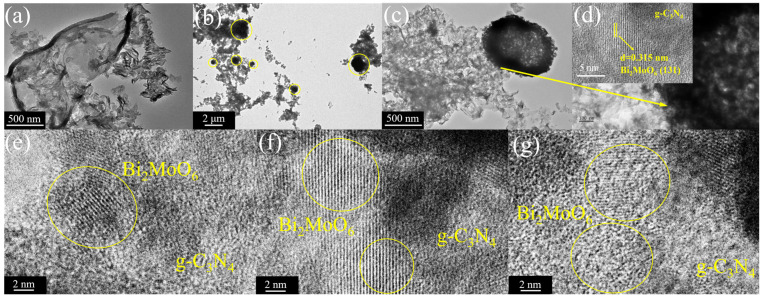
TEM and HRTEM images of OCN (**a**), P-Bi_2_MoO_6_/OCN (**b**–**d**); HRTEM images of lattice defects in P-Bi_2_MoO_6_/OCN (**e**–**g**).

The morphology of the samples was characterized by scanning electron microscopy (SEM) and transmission electron microscopy (TEM). As shown in Figure 2a,b, it can be observed that OCN is irregularly folded together by thin, few-layer, large-area nanosheets and only modified with small curved nanosheets at some endpoints or staggered positions. Figure 3c–g depict the SEM images of P-, Na- and N-doped Bi_2_MoO_6_ nanoflowers. Different states of nanoflowers can be observed; however, they have a common feature: the surface of Bi_2_MoO_6_ nanoflowers is composed of densely packed, fine nanosheet unit structures. In contrast, P-Bi_2_MoO_6_ exhibits more cracked, hollow nanoflowers, resulting in an increase in the total surface area after cracking. This is consistent with the results of XRD peaks, indicating that P-Bi_2_MoO_6_ has a larger interplanar spacing and more lattice defects and distortions. From Figure 3c–g, it can be inferred that after the formation process is completed, the internal structure of Bi_2_MoO_6_ nanoflowers has collapsed to varying degrees, resulting in the formation of hollow structures to varying degrees. Among them, the collapse rate of P-Bi_2_MoO_6_ is significantly faster than that of sodium-doped and nitrogen-doped Bi_2_MoO_6_, which may be related to the lower pH value caused by phosphomolybdic acid (H_3_PO_4_·12MoO_3_). However, the collapse modes of these three materials are similar. They all pierce from the outermost layer to the inner layer and then gradually expand, eventually forming a hollow sphere structure with a rough inner surface and fine pore distribution. This special structure is conducive to multiple scattering and slow photon effect in the process of illumination, so that more light energy can be captured and collected [4].

From Figure 2h,i, it can be inferred that after the formation process is completed, the internal structures of the Bi_2_MoO_6_ nanoflowers have collapsed to varying degrees, resulting in the formation of hollow structures to varying degrees. This is the SEM image and corresponding element mapping of OCN and P-Bi_2_MoO_6_ in situ composites. There are two composite structures of complete P-Bi_2_MoO_6_ nanoflowers and highly collapsed P-Bi_2_MoO_6_ nanoflowers hollow spheres. It can be seen that after solvent heating, the original large-area OCN nanosheet structure was destroyed and turned into a compressed small and irregularly staggered structure. From this point of view, OCN is not suitable as an improved method for long-term solvothermal heating in situ growth. In addition, in the element mapping diagram of P-Bi_2_MoO_6_/OCN, the signal intensities of N, O, Mo, Bi and P elements are represented by different colors. It can be seen that P is uniformly distributed in Bi_2_MoO_6_. In summary, through SEM and element mapping, it is effectively proven that P-Bi_2_MoO_6_ is successfully combined with OCN.

Figure 3a is the TEM image of OCN, and the transparent nanosheet image similar to the background color again proves the successful synthesis of g-C_3_N_4_ with the large area and less-thin layer. Figure 3b,c show the interface combination of P-Bi_2_MoO_6_ and OCN. Due to the existence of the internal hollow structure, the center color of P-Bi_2_MoO_6_ is lighter. In addition, there are also tiny, layered units of P-Bi_2_MoO_6_ nanoflowers on OCN nanosheets. Compared with pure OCN, the surface is rougher, and the structure is more complex, indicating that the interface between P-Bi_2_MoO_6_ and OCN is more fully bonded, which promotes the separation and transfer of carriers in the heterogeneous junction. The comparison of OCN in Figure 3a–c also clearly shows that large-area nanosheets shrink into small and tortuous structures. The HRTEM image in Figure 3d–g further proves the tightly bonded interface structure between the two. The spherical structure in the yellow circle in Figure 3b is P-Bi_2_MoO_6_, which has less light transmission than the few-layer OCN, so it is represented in the form of superimposed black shadows in the low magnification image. P-Bi_2_MoO_6_ mainly binds to OCN in the (131) crystal plane [32]. Due to the thin grain size of OCN, no obvious lattice fringes can be seen under the 2 nm high resolution transmission electron microscope. In addition, HRTEM images show the irregular and disordered lattice fringes (yellow circle out part) of P-Bi_2_MoO_6_ at the interface with OCN, which is due to lattice defects caused by P doping. These defects can effectively capture the photogenerated electrons in OCN, thereby inhibiting the recombination of excitons, enhancing the production of free radicals and ultimately improving the photocatalytic performance.

The results of XRD and SEM confirmed the simultaneous existence and close combination of the two materials. At the beginning, a large area of few-layer OCN nanosheet powder was dispersed in the synthesis solution of P-Bi_2_MoO_6_. After the beginning of the solvothermal reaction, P-Bi_2_MoO_6_ nanoflowers were gradually grown on OCN nanosheets as the substrate in a high-pressure reactor at 160 °C. Both of them are two-dimensional layered materials that are conducive to the superposition of [MoO_4_]^2−^ and [Bi_2_O_2_]^2+^ layers. Finally, with the increase of reaction time, the two are closely combined. At the same time, the shrinkage of OCN nanosheets and the hollow collapse of P-Bi_2_MoO_6_ nanoflowers appear, thus obtaining the composite materials with complex structure.

**Figure 4:**
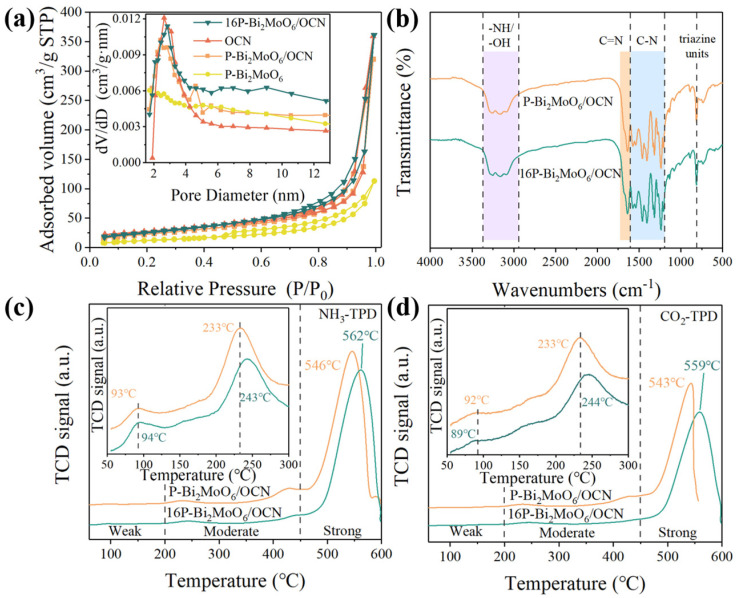
(**a**) N_2_ adsorption–desorption isotherms and BJH pore size distribution curves (inset); (**b**) FT-IR spectra of P-Bi_2_MoO_6_/OCN and 16P-Bi_2_MoO_6_/OCN; (**c**) NH_3_-TPD profile; (**d**) CO_2_-TPD profile.

As shown in Figure 4a, the specific surface area and pore structure of the OCN, P-Bi_2_MoO_6_, P-Bi_2_MoO_6_/OCN and 16P-Bi_2_MoO_6_/OCN composites were studied by N_2_ adsorption–desorption isotherm analysis. All materials exhibit distinct IV-type isotherm [33,34], indicating that they had typical layered structure and mesoporous characteristics. The specific surface area of P-Bi_2_MoO_6_/OCN is 94.78 m^2^/g (Table 2), which is significantly higher than that of P-Bi_2_MoO_6_ (46.89 m^2^/g) and P-Bi_2_MoO_6_/OCN (85.54 m^2^/g). This is due to the fact that the solvothermal treatment destroys the original structure of OCN to a certain extent. In addition to the direct observation in the SEM and TEM characterization results, the BJH pore size distribution curve is drawn. Compared with OCN and P-Bi_2_MoO_6_ monomers, the pore size distribution of P-Bi_2_MoO_6_/OCN has an additional protrusion at 4~6 nm, which is also evidence of structural changes. It is worth noting that the 16P-Bi_2_MoO_6_/OCN optimized by adding phosphoric acid does not have this protrusion, but the curve strength in the range of 4~12 nm increases uniformly, which indicates that the structure of OCN in 16P-Bi_2_MoO_6_/OCN changes more. In general, 16P-Bi_2_MoO_6_/OCN has the largest specific surface area and pore volume (97.10 m^2^/g), as well as a smaller average pore size, which is related to the fact that H_3_PO_4_ promotes the formation of more etching channels and porous structures at high temperatures, which provides more active sites for the adsorption of reactants [34]. In order to further confirm the structure of 16P-Bi_2_MoO_6_/OCN optimized by adding phosphoric acid, we compared the Fourier transform infrared spectroscopy (FT-IR) of P-Bi_2_MoO_6_/OCN and 16P-Bi_2_MoO_6_/OCN. As shown in Figure 4b, the broad peaks of the two spectra between 3364 and 2940 cm^−1^ are attributed to -NH and -OH bonds, which are related to the surface amino groups and adsorbed water molecules [35]. The peak at about 1637 cm^−1^ is the C=N bond of the g-C_3_N_4_ host. The absorption band in the range of 1600~1200 cm^−1^ is the stretching vibration peak of the aromatic C-N heterocycle. The peak at 810 cm^−1^ is a unique vibration peak of the tri-s-triazine ring system [36]. The peak observed in the range of 770–560 cm^−1^ belongs to the special absorption band of Bi_2_MoO_6_ [37]. Comparing the two spectra, it was found that the addition of phosphoric acid did not affect the characteristic structure of P-Bi_2_MoO_6_/OCN.

In order to better understand the catalytic nature, the surface acidity and basicity of P-Bi_2_MoO_6_/OCN and 16P-Bi_2_MoO_6_/OCN were evaluated by NH_3_ and CO_2_-TPD tests and were divided into three acid/basic site regions according to temperature [38,39]. The higher the desorption temperature of NH_3_ and CO_2_, the stronger the acidity and alkalinity, the larger the area of desorption peak and the greater the number of acid/base sites. The acidity and alkalinity of the material (Table 3 and Table 4) can be calculated by integrating the TPD response peaks. It can be seen from Figure 4c,d that the weak acidic and weak alkaline sites of the two materials are not obvious, and the desorption temperatures of the two materials are not much different, which indicates that the weak physical adsorption of CO_2_ and NH_3_ by the materials is less, mainly chemical adsorption. The number of weak intermediate acidic and weak intermediate alkaline sites of 16P-Bi_2_MoO_6_/OCN is lower than that of Bi_2_MoO_6_/OCN, but it has more strong acidic and strong alkaline sites. Especially, the strong alkaline sites are 16.49155 mmol/g, which is significantly higher than that of Bi_2_MoO_6_/OCN: 9.35347 mmol/g. In addition, the desorption temperature of 16P-Bi_2_MoO_6_/OCN for CO_2_ and NH_3_ is significantly higher than that of P-Bi_2_MoO_6_/OCN, which indicates that the acidic and alkaline strength of its active sites are higher. The addition of phosphoric acid changed the number and strength of acid/base sites, which may be attributed to the induced bismuth oxides such as Bi_2_O_3_ and the change of nitrogen-containing bases in OCN [40,41]. Therefore, the catalytic activity of 16P-Bi_2_MoO_6_/OCN is stronger, which may be related to the enriched basic sites on its surface. A number of studies have shown that acid/base sites are important reasons for the adsorption of water molecules on the catalyst surface, such as the formation of hydrogen bond adsorption mode, which can promote h^+^ to activate water molecules to generate more free ·OH radicals, thereby improving photocatalytic performance [42].

**Figure 5:**
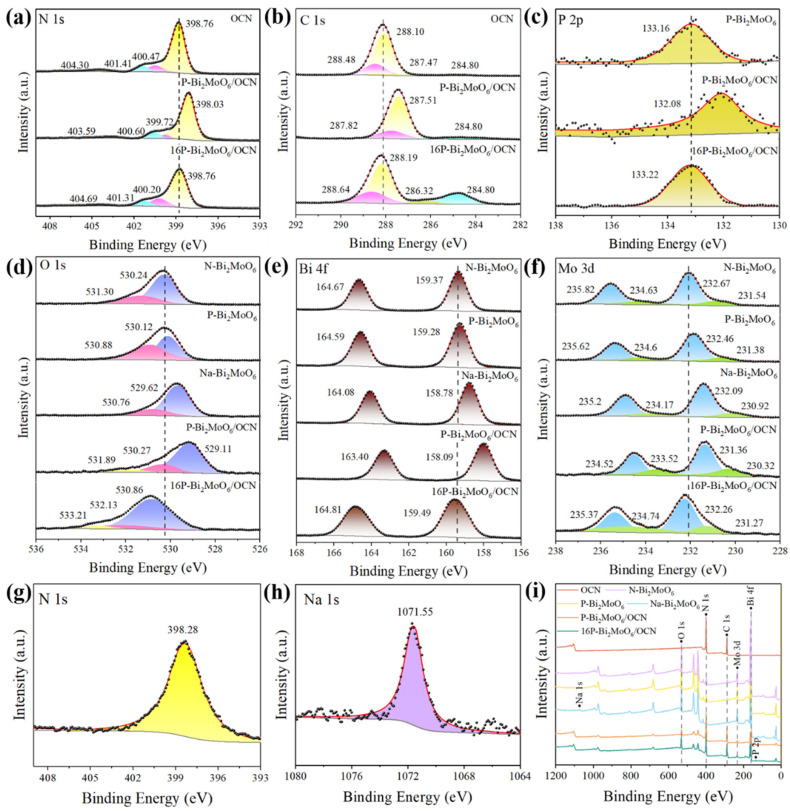
XPS spectra of N 1s (**a**,**g**), C 1s (**b**), P (**c**), O 1s (**d**), Bi 4f (**e**), Mo 3d (**f**), Na 1s (**h**) and total spectrum (**i**) of the as-synthesized samples.

The XPS spectra were used to characterize the chemical bond state on the surface of the prepared material. Figure 5 shows the comparison of XPS spectra of P, Na and N doped Bi_2_MoO_6_, OCN, P-Bi_2_MoO_6_/OCN and 16P-Bi_2_MoO_6_/OCN heterojunctions. Figure 5a shows the N 1s spectra of OCN, P-Bi_2_MoO_6_/OCN and 16P-Bi_2_MoO_6_/OCN. The main peak marked yellow at about 398.76 eV belongs to the C-N=C bond in the six-membered ring of g-C_3_N_4_, which belongs to sp^2^ hybridization [43,44]. The peak at about 400.47 eV represents the graphite nitrogen connected to three carbon atoms in the g-C_3_N_4_ six-membered ring [45]. The blue-labeled peak is N-H_x_ (x = 1,2) at the edge of the g-C_3_N_4_ structure [46]. The peak at about 404.30 eV (marked as green) is related to the charge effect, which is consistent with previous reports. After the combination of OCN and P-Bi_2_MoO_6_, the overall N 1s moves to the direction of low binding energy, which is due to the fact that OCN has many pyridine-N (C-N=C) with strong electron-withdrawing properties. The difference is that the proportion of pyridine-N in 16P-Bi_2_MoO_6_/OCN is significantly reduced, and the proportion of graphite-N (N-(C)_3_) and amino-N (N-H_x_) is increased. The final peak position is not much different from that of OCN and P-Bi_2_MoO_6_ monomers. It can be seen from Table 5 that the C-N=C/N-(C)_3_ of P-Bi_2_MoO_6_/OCN is significantly higher than that of OCN, but the C/N ratio of the two is basically the same, which indicates that the original tri-s-triazine structure of OCN is destroyed after solvothermal treatment, increasing the abundance of s-triazine structure. According to previous studies [47], the tri-s-triazine structure has better photocatalytic performance than the s-triazine structure, which makes it necessary to pay more attention to the control of reaction conditions when OCN is treated by solvothermal method. In addition, graphite-N and pyridine-N are considered to play a vital role in improving the catalytic performance and are important active centers. Although the size and mechanism of the contribution to the reaction have not yet been fully agreed upon, it is generally believed that graphite-N can effectively increase the free carrier density and promote charge transfer [48,49]. Amino-N also has a benign effect on the reaction and can improve the separation ability of photogenerated electrons and holes [50].

As shown in Figure 5b, the C 1s spectrum is fitted by peak separation, and 284.8 eV is the C-C bond, which is used as the standard reference carbon for XPS analysis. The main peak at about 288.10 eV (yellow marker) and the sub-peak at about 287.47 eV (green marker) are attributed to the main skeleton carbon N=C-N bond and the relatively small C-NH_x_ of g-C_3_N_4_, respectively. The peak at about 288.48 eV corresponds to the C-O bond, which is partly derived from the adsorption of CO_2_ in the air. The increase of C-C and C-O in 16P-Bi_2_MoO_6_/OCN may be due to the substitution of pyridine-N by C and O atoms. Figure 5d compares the O 1s spectra of N-Bi_2_MoO_6_, P-Bi_2_MoO_6_, Na-Bi_2_MoO_6_ and 16P-Bi_2_MoO_6_/OCN. The peak at about 529~532 eV belongs to the lattice oxygen, and the main peak marked as purple corresponds to the Bi-O bond in Bi_2_MoO_6_. The sub-peaks near 530~531 eV (marked as pink) are attributed to the Mo-O bond [26,51]. The small peak near the lattice oxygen is surface adsorbed oxygen, which is marked as yellow. The surface oxygen is related to the adsorption of water molecules in the air after Bi_2_MoO_6_ composite OCN, indicating the existence of O-H bond. From Table 6, it can be seen that the Bi/Mo of P-Bi_2_MoO_6_ and 16 P-Bi_2_MoO_6_/OCN is significantly higher than that of the other materials, but the O-Bi/O-Mo on the surface is significantly lower, which indicates that P doping will lead to the generation of a large number of Bi vacancies and the accumulation of surface metal Bi (Bi^0^). More lattice defects also correspond to the characteristics of the microstructure of P-Bi_2_MoO_6_ in the SEM test, that is, more collapsing of nanoflowers at the same reaction time. Compared with P-Bi_2_MoO_6_, the increase of O-Bi/O-Mo in 16P-Bi_2_MoO_6_/OCN may be attributed to the production of other Bi oxides, which would be consistent with the results of XRD. Bi vacancies have been shown to effectively promote charge transfer and separation by temporarily trapping carriers [51,52,53], thereby improving photocatalytic performance. At the same time, the metal Bi formed in situ on the surface can further promote the transfer of charge and can be used as an active center for the formation of ·OH [54]. This paper provides a method for constructing a large number of bismuth vacancies in Bi_2_MoO_6_ materials and the synergistic effect of in situ metal bismuth.

As shown in Figure 5f, the Mo 3d peak shows a pair of main peaks at 235 eV and 232 eV, indicating that it mainly exists in the form of Mo^6+^, and there are a pair of secondary peaks at about 234 eV and 231 eV, representing Mo^4+^ [55,56]. Obviously, compared with P and N doped Bi_2_MoO_6_, the Mo 3d peak of Na doped Bi_2_MoO_6_ has a more obvious right shift, which is due to the fact that Na ions have more difficulty obtaining electrons, so that the charge shifts more to Mo ions; thus, the binding energy is reduced. After the complexation of P-Bi_2_MoO_6_ with OCN, the peak position of Mo moves to a lower binding energy, which is attributed to the fact that Mo^6+^ captures the electron cloud after combining with the conjugated structure interface of OCN, which promotes the conversion of some Mo^6+^ to Mo^4+^, and the peak area ratio representing Mo^4+^ increases significantly. The proportion of Mo^4+^ in several materials is shown in Table 6. In the solvothermal process of preparing 16P-Bi_2_MoO_6_/OCN, the addition of phosphoric acid promotes the reduction of lattice bismuth to precipitate surface metal Bi, and the pyridine-N in OCN is attacked and destroyed, which are the reasons for reducing the proportion of Mo^4+^. The XPS spectrum of P in Figure 5c proves the successful introduction of P, and the peak around 133 eV is attributed to phosphates. Therefore, P tends to replace Mo in the form of P^5+^ and embed into the Bi_2_MoO_6_ lattice or interlayer gap. The N peak at 398.28 eV in Figure 5g shows that in the process of preparing N-Bi_2_MoO_6_, N is introduced in the form of carbon nitride as impurity doping. The characteristic peak of Na 1s at 1071.55 eV in Figure 5h indicates that Na was successfully doped in Na-Bi_2_MoO_6_ [57].

### 3.2. Optical Absorption Property Analysis

**Figure 6:**
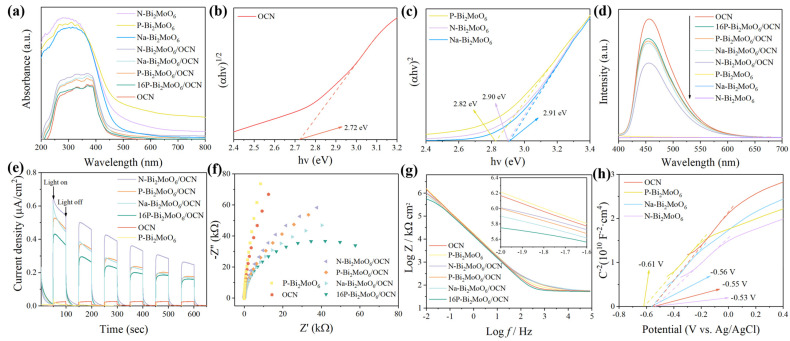
The UV-Vis DRS spectra (**a**) and the estimated band gaps of the prepared samples (**b**,**c**); PL spectra (**d**); the transient photocurrent response of the samples under intermittent visible light (**e**); the EIS plots of the samples under visible light (**f**); Bode plot (**g**); Mott–Schottky plots of the samples in the dark (**h**).

Figure 6a shows the UV-Vis DRS of a series of samples. All three doping types of Bi_2_MoO_6_ demonstrate high absorption strength within the 200~800 nm range. Among them, the absorption edge of Na-Bi_2_MoO_6_ is close to 480 nm, and the absorption edge of P-Bi_2_MoO_6_ is close to 500 nm, which is much larger than the absorption edge of OCN (around 430 nm). The coupling of OCN with Bi_2_MoO_6_ improves its light absorption ability in the visible region. As shown in Figure 6b,c, the Kubelka–Munk transformation function is used to evaluate the optical band gap (E_g_) of the prepared material:

(3) $${\left(\alpha h\nu \right)}^{\frac{1}{n}}A=\left(h\nu -E_{g}\right)$$

Bi_2_MoO_6_ is an indirect semiconductor, and g-C_3_N_4_ is a direct semiconductor. According to the type of semiconductor, the n value is different. The results are shown in Figure 6b,c. The band gap of OCN is 2.72 eV, and the band gap of Bi_2_MoO_6_ is 2.82~2.91 eV, which are similar to those reported in the literature [21,58]. The band gap of N-Bi_2_MoO_6_ is similar to that of Na-Bi_2_MoO_6_; they are 2.90 eV and 2.91 eV, respectively, but the band gap of P-Bi_2_MoO_6_ is narrower (2.82 eV). This may be due to the more lattice distortion caused by P doping, which adjusts the electronic structure and narrows the band gap of Bi_2_MoO_6_.

Figure 6d shows the photoluminescence spectra of samples such as OCN, P-Bi_2_MoO_6_, P-Bi_2_MoO_6_/OCN and 16P-Bi_2_MoO_6_/OCN to evaluate the recombination efficiency of photogenerated charge carriers. Generally, the stronger the peak, the easier it is for photogenerated electron–hole pairs to recombine. The fluorescence radiation intensity emitted by carrier recombination of Bi_2_MoO_6_ monomer is the lowest, which may be due to the fact that the absorbed photon energy is mostly released through nonradiative recombination, such as vibration, regardless of the type of doping element. Therefore, the photocatalytic degradation ability, antibacterial performance and photocurrent density of Bi_2_MoO_6_ itself are not significant. Pure OCN exhibits higher emission intensity, attributed to the easier recombination of photogenerated electron–hole pairs during photoexcitation. After compounding with Bi_2_MoO_6_, the doping types are different, showing different degrees of inhibition of carrier recombination. Among them, the fluorescence emission intensity of 16P-Bi_2_MoO_6_/OCN and P-Bi_2_MoO_6_/OCN is higher than that of other composite materials, P doping produces more Bi vacancies, and the capture effect of h^+^ makes the PL peak higher [52]. In addition, the fluorescence intensity of N-Bi_2_MoO_6_/OCN is significantly lower than that of other doped composites. This may be due to the heterojunction band gap structure formed by N-Bi_2_MoO_6_ and OCN, which is more conducive to charge transfer and makes it more effective in photocatalytic reaction.

In order to further evaluate the reactivity of the material and analyze the internal reasons for the improvement of performance, the instantaneous photocurrent curve under visible light and the electrochemical impedance spectroscopy (EIS) under illumination were measured by photoelectric measurement technology. As shown in Figure 6e, N-Bi_2_MoO_6_/OCN shows higher photocurrent intensity. The photocurrent intensity of P and Na doped Bi_2_MoO_6_/OCN composites is similar. The photocurrent response ability of 16P-Bi_2_MoO_6_/OCN decreases slightly, but all composites show stronger photoelectric conversion ability than OCN, which is consistent with the law of photoluminescence spectrum. In Figure 6f,g, the combination with the two-dimensional layered material Bi_2_MoO_6_ is obviously beneficial to reduce the impedance of OCN, and the cyclic transformation of Mo^6+^/Mo^4+^ plays an important role [59]. Compared with different doping types, sodium doped Bi_2_MoO_6_ has more advantages in promoting charge transfer than N and P doping. When studying the metal–organic interface, Blowey et al. found that alkali doping can form a two-dimensional charge transfer salt with high binding energy and increase the transfer rate by adjusting the charge barrier [60]. Therefore, Na doping can promote the charge transfer rate at the interface between Bi and Mo metal ions and OCN by acting as a connection node with special strong charge transfer ability. However, in general, the optimized 16-Bi_2_MoO_6_/OCN exhibits the smallest semicircle radius and the smallest impedance value in the low frequency region (0.01 Hz), indicating that the addition of phosphoric acid can effectively improve the charge transfer efficiency in P-Bi_2_MoO_6_/OCN, which is related to the increase of the content of surface metal Bi and the significant increase of the proportion of graphite-N in the OCN structure. As shown in Figure 6h, the flat band potentials of OCN and three doped Bi_2_MoO_6_ monomers were calculated according to the Mott–Schottky curve. The slope of the curves of several materials is positive, indicating that they are n-type semiconductors. In summary, P, Na and N doping have their own advantages, but in the actual photocatalytic reaction, they are affected by various factors. How to rationally modify and construct photocatalytic materials to improve their comprehensive performance is still a complex process that needs to be continuously explored.

### 3.3. Photo-Generated Free Radical Analysis

**Figure 7:**
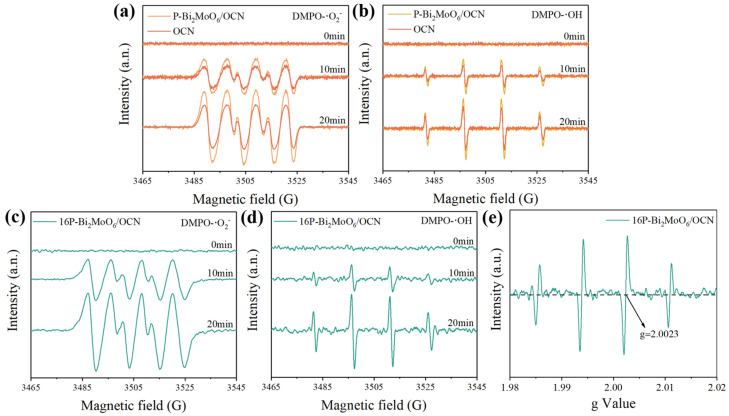
EPR signals of DMPO-·O_2_^−^ (**a**,**c**) and DMPO-·OH (**b**,**d**) for the samples. EPR spectra of the oxygen vacancy in 16P-Bi_2_MoO_6_/OCN (**e**).

The role of active free radicals in the photocatalytic process was discussed by using EPR technology. Figure 7 shows the free radical paramagnetic response of OCN and P-Bi_2_MoO_6_ under the same xenon lamp. There is no paramagnetic response in the dark state (time 0), and then it is illuminated with a xenon lamp equipped with a 420 nm filter and tested every 10 min. According to the flat band potential determined by the electrochemical Mott–Schottky curve and the band gap width obtained by the Kubelka–Munk transform function, the band gap structure fitted indicates that OCN has the thermodynamic condition for photocatalytic generation of ·OH and ·O_2_^−^. This is because after forming heterojunctions with P-Bi_2_MoO_6_, OCN and P-Bi_2_MoO_6_, the lattice defects caused by P doping promote the separation and transfer of photogenerated carriers, promote the generation of more free radicals and improve the photocatalytic performance. After adding an appropriate amount of phosphoric acid, 16P-Bi_2_MoO_6_/OCN can not only achieve faster charge transfer and more surface reactive active sites but also have a band gap structure that can produce two free radicals, with strong DMPO-·O_2_^−^ and DMPO-·OH signal values (Figure 7c,d). In addition, the g value of EPR signal is 2.0023, which indicates that there are a large number of free electrons and oxygen vacancies in 16P-Bi_2_MoO_6_/OCN [61].

**Figure 8:**
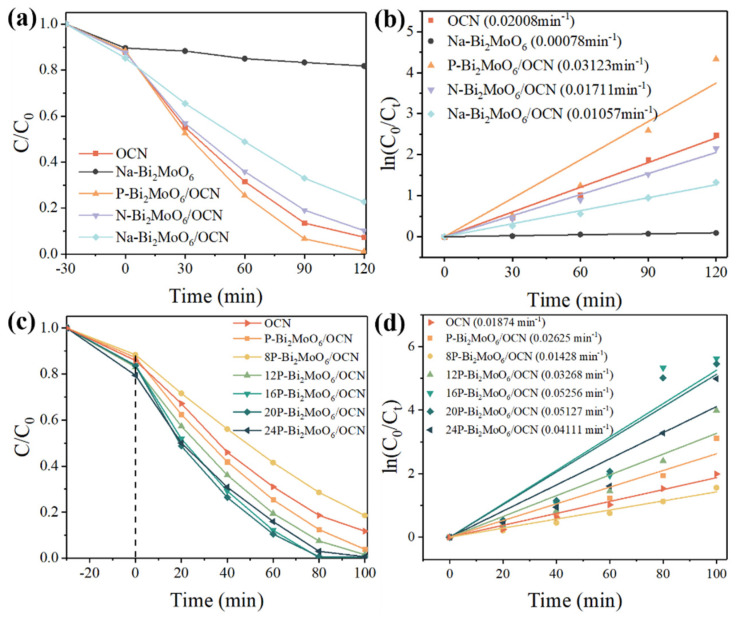
The photocatalytic degradation rate of RhB solution of the samples under visible light irradiation (**a**,**c**) and the corresponding first-order kinetic plots (**b**,**d**).

Figure 8 shows the visible light degradation results of 20 mg/L RhB (50 mL) by the heterojunction composed of OCN and three doping types of Bi_2_MoO_6_ at 25 mg catalyst dosage. Na-Bi_2_MoO_6_ was used as the representative material for the degradation experiment of Bi_2_MoO_6_ monomer. This is because the three doping types have little difference in the characteristics of optical characterization. The results show that Bi_2_MoO_6_ monomer has almost no degradation performance under visible light. This is due to the low quantum yield and low activity of Bi_2_MoO_6_. In the previous literature, pure Bi_2_MoO_6_ does not show obvious photocatalytic performance [62,63]. Compared with different doping, P-Bi_2_MoO_6_/OCN showed the highest photocatalytic degradation performance, and the degradation rate of 20 mg/L RhB was 98.8% within 2 h.

It is worth noting that although N-Bi_2_MoO_6_/OCN and Na-Bi_2_MoO_6_/OCN exhibit low peak intensity in photoluminescence spectra, their actual photocatalytic degradation performance is lower than that of pure OCN. This is not only because the specific surface area of Bi_2_MoO_6_/OCN doped with Na and N is lower than that of OCN, which is conducive to the lack of active sites adsorbed by the reactants, so that the photogenerated carriers cannot play the role of oxidation and reduction faster, limiting the photocatalytic degradation rate. At the same time, the structure of OCN is destroyed after solvothermal treatment, which reduces the abundance of tri-s-triazine structure, which is more conducive to photocatalytic reaction. These factors make OCN have higher requirements for composite materials. The difference is that due to the more lattice defects of P-Bi_2_MoO_6_, a large number of Bi vacancies can promote the transfer of photogenerated carriers by capturing the carriers of OCN and coordinating with the in situ metal Bi. In addition, as a dopant and reaction source, phosphomolybdate itself has abundant Lewis and Brønsted acid sites. Many studies have shown that the introduction of P can produce strong Lewis and Brønsted acid sites, especially highly active Lewis acid sites [64], which interact with strong Lewis base OCN to increase the adsorption and activation of O_2_ and other reactants, thereby improving photocatalytic performance. A series of optimized P-Bi_2_MoO_6_/OCN were obtained by adding different amounts of phosphoric acid to increase the P doping content in the reaction, and the degradation performance of 20 ppm RhB with the same amount of catalyst was tested. It should be noted that, taking OCN as an example, the rate constant (k) obtained by fitting the degradation kinetics of Figure 8d was slightly lower than that of the previous group of experiments (Figure 8b), which is a systematic error and environmental reason such as the decrease of light intensity caused by the lifetime loss of xenon lamp. After optimization, 16P-Bi_2_MoO_6_/OCN had the best degradation performance, and the dye was completely degraded within 80 min. This shows that after optimization, the improvement of the specific surface area of the material, the improvement of the charge transfer rate and more active sites effectively improve the photocatalytic performance.

**Figure 9:**
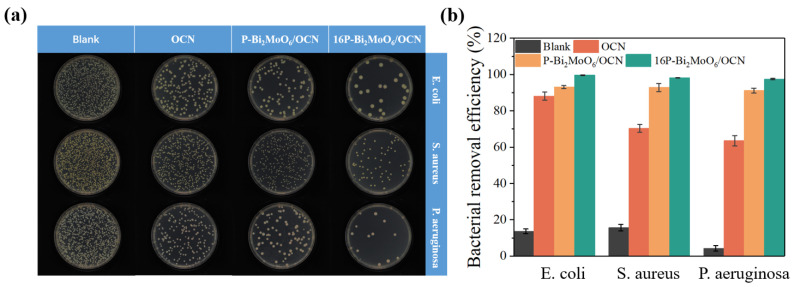
(**a**) The colony image of the antibacterial experiment coated on agar plates after 300 min of visible light illumination; (**b**) after 300 min of visible light illumination, the photocatalytic sterilization rate of the material on *E. coli* (around 2.5 × 10^6^ CFU/mL, 50 mL), *S. aureus* (around 2.0 × 10^6^ CFU/mL, 50 mL) and *P. aeruginosa* (around 2.1 × 10^6^ CFU/mL, 50 mL).

By the photocatalytic degradation of RhB, 16P-Bi_2_MoO_6_/OCN, with high photocatalytic performance, was selected for the comparative antibacterial test. Figure 9a,b show the plate antibacterial experiments and antibacterial rate comparison of *E. coli*, *S. aureus* and *P. aeruginosa* to evaluate their photocatalytic, broad-spectrum antibacterial properties under visible light. The experimental conditions such as the light conditions and catalyst delivery quality of each group of antibacterial experiments are the same. In the blank sample without catalyst input, the number of colonies did not decrease significantly after 300 min of illumination, indicating that visible light had little effect on *E. coli*, *S. aureus* or *P. aeruginosa* in this experiment. The antibacterial rates of 16P-Bi_2_MoO_6_/OCN against *E. coli*, *S. aureus* and *P. aeruginosa* in 300 min were 99.58%, 98.20% and 97.48%, respectively, which were significantly higher than those of OCN against the three bacteria (88.09%, 70.31% and 63.55%, respectively). Compared with P-Bi_2_MoO_6_/OCN, the antibacterial properties of 16P-Bi_2_MoO_6_/OCN were further improved. The composite of P-Bi_2_MoO_6_ and OCN effectively suppressed the recombination of photogenerated carriers and made carriers more efficiently transfer to the surface of the material to participate in the reaction. The capture of Bi vacancies caused by P doping, as well as high active sites such as surface metal Bi and acid/base sites, was conducive to consuming O_2_ and other substances, producing more reactive oxygen radicals, oxidizing and attacking bacterial cell membranes and cell walls and leading to bacterial rupture and death. The addition of phosphoric acid-optimized 16P-Bi_2_MoO_6_/OCN induced the regulation of the nitrogen ratio of OCN and increased the basic active sites and surface metal Bi, which was more conducive to the adsorption and activation of reactants. Finally, the photocatalytic performance was significantly improved by fine-tuning the P doping content. In addition, the electron transfer between Mo^6+^ and Mo^4+^ in P-Bi_2_MoO_6_/OCN also accelerated the charge conduction. Several studies have shown that the redox cycle reaction between Mo^6+^ and Mo^4+^ continuously stimulates more ROS production [59,65,66].

**Figure 10:**
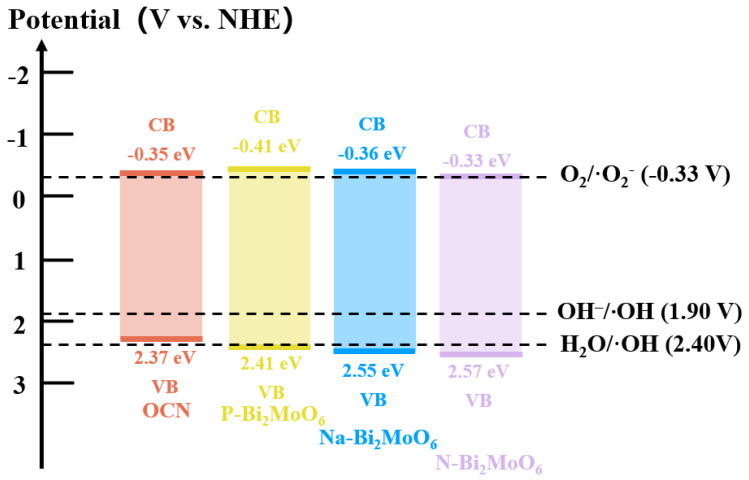
The schematic of energy band alignments for the prepared OCN, P-Bi_2_MoO_6_, Na-Bi_2_MoO_6_ and N-Bi_2_MoO_6_.

Through a series of characterization experiments, as well as the degradation experiments of RhB under visible light and the antibacterial experiments of three strains, the photocatalytic reaction mechanism of P-Bi_2_MoO_6_/OCN was proposed. Firstly, the band gap structure relationship of P-Bi_2_MoO_6_/OCN heterojunction was obtained by UV-Vis DRS and electrochemical Mott–Schottky curve. It should be noted that on the basis of the flat band potential obtained by Ag/AgCl reference, 0.199 eV was added to convert into a standard hydrogen electrode as a reference, and the band structure relationship shown in Figure 10 was obtained after fitting. It can be seen from the energy band relationship that the conduction band and valence band of OCN were contained in the band gap of P-Bi_2_MoO_6_ and Na-Bi_2_MoO_6_, which is a typical feature of type I heterojunction, but it was a type II heterojunction with staggered band gap with Na-Bi_2_MoO_6_. Under light excitation, the electrons on the valence band of P-Bi_2_MoO_6_ absorbed light energy, activated the transition to the conduction band and then released the energy back to the conduction band of OCN with a corrected potential to generate free radicals to participate in the redox reaction. Although the quantum yield of photoexcited P-Bi_2_MoO_6_ was very low, so that it could only transfer few photogenerated electrons and holes to OCN in photocatalytic reaction, the photoluminescence, photo electrochemistry, degradation and antibacterial results under visible light all proved that the composite material significantly inhibits the recombination rate of photogenerated carriers and has higher photocatalytic performance. Based on the above results, this indicates that the P-Bi_2_MoO_6_/OCN heterojunction not only improves the performance by constructing the traditional charge transfer form of type I heterojunction. Considering that P doping can endow Bi_2_MoO_6_ with abundant Lewis acid sites and Brønsted acid sites, the former (Lewis acid sites) especially have higher activity and electron absorption capacity, while OCN itself has strong Lewis basic sites, both of which form sterically hindered Lewis bases and Lewis acids, which greatly enhance the adsorption and activation of reactant molecules and improve the conversion efficiency of O_2_ to ·O_2_^−^ [42,67,68]. At the same time, the lattice defects (Bi^3+^ vacancies) caused by P doping are greater than the other two doping types, which tend to capture the holes of OCN as the active sites for the generation of ·OH and cooperate with the in situ metal Bi to promote the separation and transfer of photogenerated charge carriers. In addition, the cyclic transformation of Mo^6+^ and Mo^4+^ in P-Bi_2_MoO_6_ provides more opportunities for electron transfer, so the electrons consumed on the surface of P-Bi_2_MoO_6_ can also be supplemented by rapid interlayer transfer. After re-optimization, 16P-Bi_2_MoO_6_/OCN with higher P doping amount was obtained. The phosphoric acid etching effect increased the specific surface area and induced the increase of the proportion of graphite nitrogen on OCN and the precipitation of Bi_2_MoO_6_. More metal Bi and related oxides made the adjusted structure more conducive to charge separation and transfer. Compared with other doping types of Bi_2_MoO_6_/OCN, the band gap structure of Na-Bi_2_MoO_6_/OCN conforms to the type I heterojunction. However, in addition to the faster charge transfer rate, there is no other mechanism that can further improve the photocatalytic performance. N-Bi_2_MoO_6_ and OCN can form a type II heterojunction, so that part of the photogenerated electrons on the OCN conduction band first fall into the conduction band of N-Bi_2_MoO_6_, reducing the recombination with holes on the valence band, thereby achieving effective separation of photogenerated carriers. However, N-Bi_2_MoO_6_/OCN also does not have enough active sites as a carrier for photocatalytic reactions, making it still unable to compensate for the performance degradation caused by structural damage of OCN after solvothermal treatment. In summary, the combination of OCN and P-Bi_2_MoO_6_ effectively inhibited the recombination of OCN photogenerated carriers. Compared with N and Na doping, it compensated for the decrease of photocatalytic activity caused by the decrease of active sites after OCN solvothermal treatment and finally achieved high photocatalytic degradation and antibacterial properties.

## 4. Conclusions

In this paper, three molybdenum sources and three doping types (P, Na and N) of Bi_2_MoO_6_ nanoflowers were successfully designed and synthesized for in situ growth on g-C_3_N_4_ nanosheets. The effects of different molybdenum sources and corresponding doping on the photocatalytic performance of doped Bi_2_MoO_6_/g-C_3_N_4_ composite system were studied.

1. Firstly, by comparing the composite of Bi_2_MoO_6_ with different doping elements grown on the surface of OCN under the same conditions, it was found that after compounding, the OCN structure collapsed and shrunk into small, interlaced g-C_3_N_4_ nanosheets, and the structure was destroyed, which increased the s-triazine structure, but the photocatalytic performance was low and brought disadvantages.
2. Furthermore, it is worth noting that in Bi_2_MoO_6_ prepared from different molybdenum sources, compared with OCN/P or Na or N doped Bi_2_MoO_6_, it is found that the collapsed P-Bi_2_MoO_6_/OCN still shows further improved performance compared with Na-Bi_2_MoO_6_/OCN and N-Bi_2_MoO_6_/OCN, which is significantly higher than any other system. This is because P doping brings more lattice defects and active sites, which helps to promote the separation of photogenerated carriers and the adsorption and activation of reactants. Due to the introduction of abundant Lewis acid sites and other highly active reaction sites by phosphomolybdate, it interacts with OCN of strong Lewis base to effectively promote the generation of superoxide anion radicals by photogenerated electrons. Therefore, it effectively compensates for the unfavorable factors caused by the structural damage of OCN and further improves the photocatalytic activity of the P-Bi_2_MoO_6_/g-C_3_N_4_ system. In contrast, although the heterojunction of Na- and N-doped Bi_2_MoO_6_ prepared from sodium molybdate and ammonium molybdate as molybdenum sources with the shrunken OCN can effectively suppress the recombination of photogenerated carriers, it still reduces the photocatalytic performance due to the inability to strongly compensate for the reduction of active sites.
3. Finally, in order to further optimize, the P doping content in P-Bi_2_MoO_6_/OCN was increased. The etching effect of phosphoric acid increased the specific surface area of the material, promoted more Bi vacancies and was also accompanied by more metal Bi and Bi oxide precipitation surface, which promoted the charge transfer efficiency. At the same time, the proportion of N atom distribution in OCN was induced and regulated, and more basic sites were obtained. These factors further improve the photocatalytic performance of P-Bi_2_MoO_6_/OCN.

In summary, the optimal construction process of photocatalytic materials will be affected by various factors; in the actual photocatalytic reaction, the reaction sites with higher activity may have a more important role in promoting the photocatalytic performance. Finally, an environmentally friendly P-Bi_2_MoO_6_/g-C_3_N_4_ with high photocatalytic antibacterial activity was successfully prepared by a simple preparation method using phosphomolybdic acid as the molybdenum source and dopant. The degradation rate of 20 mg/L RhB by 16P-Bi_2_MoO_6_/OCN with the best performance after optimization was 99.7% in 80 min under visible light. The antibacterial rates of *E. coli*, *S. aureus* and *P. aeruginosa* were 99.58%, 98.20% and 97.48%, respectively, in 300 min. This provides a valuable reference for optimizing environmentally friendly and efficient photocatalytic antibacterial materials.

## Figures and Tables

**Table 1:** Cell parameter data of several Bi_2_MoO_6_ with orthorhombic crystal structure.

Sample	a (Å)	b (Å)	c (Å)	Vol (Å^3^)
P-Bi_2_MoO_6_	5.55	16.23	5.44	490.70
Na-Bi_2_MoO_6_	5.50	16.21	5.48	489.11
N-Bi_2_MoO_6_	5.53	16.14	5.49	490.38

**Table 2:** Specific surface area, pore volume and average pore radius of the as-prepared materials.

Sample	S_BET_ (m^2^/g)	Pore Volume (cm^3^/g)	Average Pore Radius (nm)
OCN	94.78	0.16	12.48
P-Bi_2_MoO_6_	46.89	0.10	4.54
P-Bi_2_MoO_6_/OCN	85.54	0.17	10.69
16P-Bi_2_MoO_6_/OCN	97.10	0.19	10.65

**Table 3:** NH_3_-TPD quantification results of the catalysts.

Sample	Quantity of Weak and Medium Acid Sites (mmol/g)	Quantity of Strong Acid Sites (mmol/g)	Total Quantity (mmol/g)
P-Bi_2_MoO_6_/OCN	0.18254	0.78667	0.96921
16P-Bi_2_MoO_6_/OCN	0.12939	0.8423	0.97169

**Table 4:** CO_2_-TPD quantification results of the catalysts.

Sample	Quantity of Weak and Medium Base Sites (mmol/g)	Quantity of Strong Base Sites (mmol/g)	Total Quantity (mmol/g)
P-Bi_2_MoO_6_/OCN	0.01025	9.34322	9.35347
16P-Bi_2_MoO_6_/OCN	0.00197	16.48958	16.49155

**Table 5:** Atomic distribution of N and C atoms based on XPS N 1s and C 1s spectra.

Sample	C-N=C	N-(C)_3_	NH_x_	C-N=C/N-(C)_3_	C/N
OCN	76.44%	9.99%	6.28%	7.65	0.45
P-Bi_2_MoO_6_/OCN	80.81%	3.05%	8.05%	26.50	0.46
16P-Bi_2_MoO_6_/OCN	57.25%	22.11%	12.45%	2.59	0.57

**Table 6:** Atomic distribution of O, Bi and Mo atoms based on XPS O 1s, Bi 4f and Mo 3d spectra.

Sample	Bi/Mo	O-Bi/O-Mo	Mo^4+^/Mo^6+^
P-Bi_2_MoO_6_	8.13	1.04	24.01
Na-Bi_2_MoO_6_	7.10	3.52	30.79
N-Bi_2_MoO_6_	6.75	2.98	27.18
P-Bi_2_MoO_6_/OCN	6.94	3.52	41.12
16P-Bi_2_MoO_6_/OCN	9.52	2.72	20.79

## Data Availability

The data are contained within the article.
